# Meningioma classification by immunohistochemistry: A replicability study

**DOI:** 10.1016/j.bas.2022.101711

**Published:** 2022-12-27

**Authors:** Olivia Näslund, Anna Lipatnikova, Anna Dénes, Cecilia Lindskog, Thomas Olsson Bontell, Anja Smits, Asgeir S. Jakola, Alba Corell

**Affiliations:** a Department of Clinical Neuroscience, Institute of Neuroscience and Physiology at the Sahlgrenska Academy, University of Gothenburg, Gothenburg, Sweden; b Department of Neurosurgery, Sahlgrenska University Hospital, Gothenburg, Sweden; c Department of Neurosurgery, St. Olavs Hospital, Trondheim University Hospital, Trondheim, Norway; d Department of Clinical Pathology, Sahlgrenska University Hospital, Gothenburg, Sweden; e Department of Physiology, Institute of Neuroscience and Physiology at the Sahlgrenska Academy, University of Gothenburg, Gothenburg, Sweden; f Sahlgrenska Academy, University of Gothenburg, Gothenburg, Sweden; g Department of Immunology, Genetics and Pathology, Uppsala University, Uppsala, Sweden; h Department of Medical Sciences, Neurology, Uppsala University, Uppsala, Sweden

**Keywords:** Meningioma, Molecular marker, Recurrence, Immunohistochemistry

## Abstract

**Introduction:**

Meningiomas account for nearly 40% of intracranial tumors. Recently, the immunohistochemistry (IHC) markers S100B, SCGN, ACADL and MCM2 have been shown to be associated with underlying biological subtypes of meningioma (MG1-MG4). We aimed to evaluate these IHC markers in a clinical setting.

**Research question:**

Are the new proposed IHC markers clinically useful?

**Methods:**

In total, 244 patients with meningiomas with tissue in TMAs were included and the IHC markers S100B, SCGN, ACADL and MCM2 were analyzed. Two sets of analyses were performed; the first included all samples with any staining considered positive, the second only samples with >10% immunopositivity. PFS and OS were analyzed in correlation to immunopositivity in the second analysis set.

**Results:**

In the first set of analyses only 26.2% of samples could be to allocate to one group. No further analyses were performed with this selection. In the second set of analyses 52.0% could be allocated to a group. There was an enrichment of WHO grade 2 and 3 tumors in MG3 and MG4 as compared to MG1 (24.1% and 25.7% vs. 12.1%). Both the molecular group (p ​= ​0.032) and WHO grade (p ​= ​0.005) had significant impact on PFS, but only WHO grade predicted OS (p ​= ​0.033).

**Conclusion:**

We studied the proposed new method of classifying meningiomas into groups MG1, MG2, MG3 and MG4 using IHC markers, but found difficulties applying the classification system in our material mainly due to lack of exclusivity of markers. Thus, in its present form the classification method lacks clinical applicability.

## Highlights

• There is a clinical need to better predict outcome in patients with meningioma.
• A classification system where IHC can be used in in group allocation has been proposed.
• The IHC classification was hampered by lack of exclusivity.
• In our study, WHO grade but not the IHC based meningioma groups, predicted overall survival.
• In its present form the IHC classification method lacks clinical applicability.

## 1 Introduction

Meningiomas make up as many as 40% of primary intracranial tumors in the adult population. Meningiomas are classified as grade 1–3 according to the World Health Organization (WHO) Classification of Tumors in the Central Nervous System (CNS) (Louis et al., 2021). The WHO classification from 2016 was the first to include molecular markers for diagnosis of intracranial tumors, such as gliomas (Louis et al., 2016). In 2021, the WHO classification was again revised with major changes, and new tumor types were introduced that exemplify the role of molecular diagnostics in CNS tumor classification (Louis et al., 2021). However, meningiomas are still considered a single tumor type with 15 subtypes based on morphological criteria. Several molecular biomarkers, among others SMARCE1, BAP1, KLF4/TRAF7, have been associated with specific subtypes of meningioma, whilst TERT promotor mutation, homozygous deletion of CDKN2A/B, and H3K27me3 loss of nuclear expression are considered associated with grading of these tumors (Louis et al., 2021).

The biological behavior of meningiomas differs between the WHO grades, but also within the different grades. It has been found that up to 25% of grade 1 meningiomas recur, even though they are anticipated to have a more indolent course of disease. Likewise, up to 71% of grade 2 meningiomas that are predicted to recur do not recur (Louis et al., 2016; Patel et al., 2019; Sahm et al., 2017). This clinical variability reflects the need of stable molecular markers for improved prognosis. There has been a surge in new techniques and algorithms for subgrouping and classifying meningiomas, which may provide diagnostic and prognostic information beyond the WHO classification (Sahm et al., 2017; Maas et al., 2021; Paramasivam et al., 2019; Mirian et al., 2020; Nassiri et al., 2019; Driver et al., 2021; Choudhury et al., 2022). As the histopathological diagnosis is associated with inter-observer variability, and not always representative of clinical course, novel and more reliable markers are of clinical interest (Saygin et al., 2020; Backer-Grøndahl et al., 2012). Of specific interest is the recent study by Nassiri et al. (2021) in which four molecular groups were identified, revealing group-specific markers through a combination of DNA somatic copy-number aberrations, DNA somatic point mutations, DNA methylation and messenger RNA abundance. These four groups, denoted MG1-MG4, were found to have an improved clinical relevance as compared to existing classification strategies. Tumors of all WHO grades were represented in MG2-MG4, whereas MG1 was only composed of WHO grade 1 and 2 meningiomas. Tumors of higher WHO grade were more frequently found in MG3 and MG4, with a decrease in recurrence-free survival time with increasing molecular group. One potential advantage of this method of classification is that simple IHC could be used with high accuracy for group assignment, a diagnostic benefit not provided by most other algorithms that are based on methylation-classification. Indeed, methylation has been found to have some inherent problematic technical factors (Capper et al., 2018; Do and Dobrovic, 2015; Ludyga et al., 2012), and as for today there is a need for methodological adjustments before this method can be applied in daily routine of the grading of meningiomas (Louis et al., 2020). Another disadvantage of methylation-based classification is that it is often limited to a small number of centers, while meningiomas occur worldwide and are often treated outside of large, academic institutions (Curry and Barker, 2009).

In this study, we aimed to test the proposed IHC-based classification of meningiomas suggested by Nassiri et al. (2021) and the association with clinical outcome in a large cohort of meningiomas.

## 2 Methods

### 2.1 Patient selection

Patients treated surgically for brain tumors between January 2004 to December 2017 ​at the Department of Neurosurgery at Sahlgrenska University hospital, Gothenburg, were included. In retrospect, we selected patients undergoing first time neurosurgical intervention for a later histologically verified meningioma, and excluded patients who had previously undergone meningioma surgery, as well as patients missing baseline variables. In total, 655 patients were identified, of which 244 patients simultaneously were included in tissue microarray (TMA) cohort and selected for further analysis.

### 2.2 Clinical characteristics

The clinical data that was extracted included the following parameters: age, gender, Karnofsky performance score (KPS) (Schag et al., 1984), tumor location, number and size, presence of radiological edema, calcification or involvement of venous sinuses, Simpson grade (Simpson, 1957), histopathological grading according to WHO at time of surgery, course of disease and adjuvant therapy, and death before end of follow up (November 15th, 2021). All patients had a minimum of four and maximum of 17 years of follow-up, and the median follow-up time was eight years. The term “progression” was used to describe: 1) progression of a known remnant, or 2) recurrence in a patient with a previous radical removal as defined by Simpson grade (grade 1–3) and postoperative brain scanning. The largest tumor diameter was measured in the sagittal, coronal, or axial plane using the most recent routinely performed preoperative magnetic resonance imaging (MRI). The location of the tumor was classified as olfactory groove, suprasellar, clivus, foramen magnum, cerebellar, parasagittal, paranasal, optic sheath, sphenoid wing, posterior fossa, tentorium falx, convexity, or intraventricular.

### 2.3 Tissue samples

Tissue samples were handled within the clinical histopathological setup at Sahlgrenska University hospital, Gothenburg, Sweden. In brief, tumor tissue was fixed with 4% formaldehyde, dehydrated, and embedded in paraffin. Paraffin blocks with representative tumor areas were then selected by a neuropathologist (T.O.B.) for each patient case and marked for two tissue cores per donor block in preparation for subsequent TMA production (A.D.). TMA production and immunohistochemical staining was performed by the Human Protein Atlas consortium (Uhlén et al., 2015) (www.proteinatlas.org), at the Tissue profiling site, Uppsala University, Sweden, as previously described (Kampf et al., 2012). To detect the proteins specifically enriched in each molecular group reported by Nassiri et al. (2021) immunohistochemical analyses were performed on TMA-sections.

### 2.4 Immunohistochemical analysis

According to the abovementioned publication, groups were associated with the following markers - S100B for MG1, SCGN for MG2, ACADL for MG3, and MCM2 for MG4 (Nassiri et al., 2021). In the present study, we used the following antibodies: anti-S100B (HPA015768, Atlas Antibodies, Bromma, Sweden, 1:5000), anti-SCGN (HPA006641, Atlas Antibodies, 1:500), anti-ACADL (HPA011990, Atlas Antibodies, 1:100), anti-MCM2 (MCA1859, Bio-Rad Antibodies, Solna, Sweden, 1:200). Images of stained TMA sections were scanned in 40x magnification using the Leica Aperio AT2 automated scanning system (Leica Biosystems, Buffalo Grove, IL, USA), and assessed in the software Aperio ImageScope (Leica Biosystems). See Supplementary Table for differences in use of antibodies compared to the publication by Nassiri et al.^13^

Each tumor was represented by two tissue cores stained for each of the four markers, and each stained tissue core was assessed on a seven-graded scale based on intensity of staining (weak or strong) and proportion of stained cells, where 0 indicated negative staining, 1 ​< ​10%, 2 10–50%, and 3 ​> ​50% positively stained cells. Analyses of the TMAs were supervised by a neuropathologist (T.O.B). Each stained core was independently annotated by two observers in a blinded fashion, and in case of uncertainties or discrepancies consensus was reached together with a neuropathologist (16/976, 1.6%).

### 2.5 Statistical analysis

Cohort size in this retrospective analysis was determined by the availability of tissue samples rather than derived from statistical power analysis. Statistical analyses were performed using the IBM SPSS version 27 software. A *p*-value of <0.05 was considered significant. All tests were two-sided, and central tendencies were presented as means ​± ​SD or median and first and third quartile if skewed. Normality was assessed using Kolmogorov-Smirnov test. Kaplan-Meier analysis with Log Rank test was used to calculate progression-free survival and overall survival.

## 3 Results

### 3.1 Clinical characteristics

**Fig. 1:**
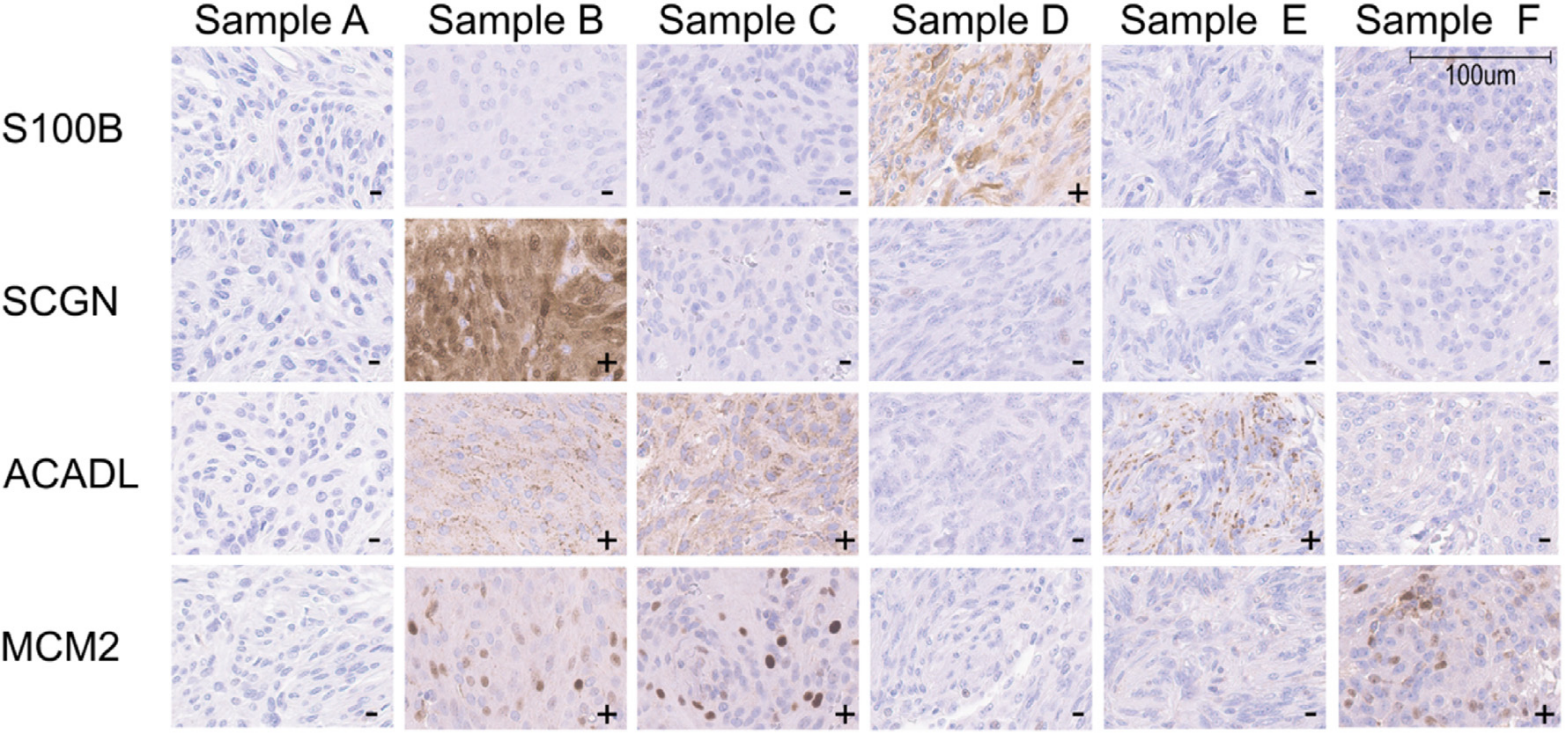
Immunohistochemical staining of S100B, SCGN, ACADL and MCM2 in six different tumor samples. *Sample A*: All negative; *Sample B*: S100B negative, the rest positive; *Sample C*: S100B and SCGN negative, ACADL and MCM2 positive; *Sample D*: S100B positive, the rest negative; *Sample E*: ACADL positive, the rest negative; *Sample F*: MCM2 positive, the rest negative.

For patient, tumor, and outcome characteristics, see Table 1. Median age at surgery was 60 years, and 67.2% were female. The most common tumor location was the convexity (n ​= ​98, 40.2%). WHO grade 1 was observed in 182 (74.6%). At the end of follow up, 67 patients (27.5%) had either recurred or experienced progression of a known remnant. Reoperation was performed in 35 patients (14.3%), whilst postoperative radiotherapy was provided in 19 patients (7.8%). Before end of follow-up, 39 patients (16.0%) of the total cohort were deceased. Fig. 1 shows immunostainings of representative cases for each of the markers, in addition to staining patterns in different tumor samples.

**Table 1:** Demographic and clinical characteristics (n ​= ​244).

Patient and tumor variables	Patients
Age at surgery, median (Q1-Q3)	60 (48–70)
Female, n (%)	164 (67.2)
Karnofsky score >70, n (%)	188 (77)

**Tumor location**	
Convexity, n (%)	98 (40.2)
Parasagittal, n (%)	18 (7.4)
Falx, n (%)	46 (18.9)
Sphenoid wing, n (%)	47 (19.3)
Other, n (%)	35 (14.3)

**Radiological variables**	
Contact or invasion of venous sinus, n (%)	120 (49.2)
Largest diameter at diagnosis in mm, mean (SD)	44.4 (16.4)
Solitary meningioma, n (%)	220 (90.2)
Edema, n (%)	157 (64.3)
Calcification, n (%)	43 (17.6)

**WHO grade**^a^	
1, n (%)	182 (74.6)
2, n (%)	53 (21.7)
3, n (%)	7 (2.9)
Missing, n (%)	2 (0.8)

**Simpson grade**	
1, n (%)	110 (45.1)
2, n (%)	113 (46.3)
3, n (%)	4 (1.6)
4, n (%)	17 (7)

**Outcome variables**	
Progression, n (%)	67 (27.5)
Reoperation, n (%)	35 (14.3)
Postoperative radiation, n (%)	19 (7.8)
Death before end of follow-up^b^, n (%)	39 (16.0)

a As graded by the classification used at time of surgery (2000, 2007 or 2016).

b November 15th, 2021

### 3.2 Classification based upon immunohistochemistry

As seen in Table 2, only 64 patients (26.2%) could initially be assigned to either one of the four molecular groups as proposed by Nassiri et al. (2021). In our material, most cases were initially classified as MG4, whilst no tumors were attributed to group MG2. Hence, 178 patients (73.8%) were considered “undecided”, with tissue staining for zero, two, or more markers, rendering these tumors impossible to attribute to a specific group. Table 3 shows the percentage distribution of stained cells in tumors successfully assigned to a molecular group. We noted that a large proportion of patients (n ​= ​30, 46.9%), assigned to group MG4, only stained very scarcely, i.e., <10%. Tumors assigned to MG4 could also often be categorized into another molecular group, and hence we chose to perform a second set of analysis where a minimum value of 10% immunopositive cells was set in order to qualify into a specific molecular group. These results are presented in Table 2, column two, showing a different distribution, with a larger proportion of tumors stained positive for one marker (52.0% vs 26.2%) that thus could be assigned to a group.

**Table 2:** Marker characteristics. In column one, all stains were considered positive regardless the staining percentage, whilst in column two all stains<10% were analyzed as negative (n ​= ​244).

Markers	Any staining considered positive	0-< 10% staining considered negative
**No positive markers**, n (%)	4 (1.6)	52 (21.3)

**One positive marker**, n (%)	64 (26.2)	127 (52.0)
MG1, n (%)	2 (0.8)	33 (13.5)
MG2, n (%)	0 (0)	1 (0.4)
MG3, n (%)	7 (2.9)	58 (23.8)
MG4, n (%)	55 (22.5)	35 (14.3)

**Two positive markers**	132 (54.1)	58 (23.8)
MG1+MG2, n (%)	0 (0)	0 (0)
MG1+MG3, n (%)	1 (0.8)	8 (13.4)
MG1+MG4, n (%)	32 (24.2)	10 (17.2)
MG2+MG3, n (%)	0 (0)	0 (0)
MG2+MG4, n (%)	10 (7.6)	0 (0)
MG3+MG4, n (%)	89 (67.4)	40 (70.0)

**Three positive markers**, n (%)	40 (16.3)	5 (2.0)
**Four positive markers**, n (%)	2 (0.8)	0 (0)
**Missing**, n (%)	2 (0.8)	2 (0.8)

**Table 3:** Percentage of stained cells in patients successfully assigned to only one molecular group based on the first set (any staining considered positive) (n ​= ​64).

Percentage of stained cells	<10%	10–50%	>50%
**MG1,** n (%)	0 (0)	1 (1.6)	1 (1.6)
**MG2**, n (%)	0 (0)	0 (0)	0 (0)
**MG3**, n (%)	1 (1.6)	4 (6.3)	2 (3.1)
**MG4**, n (%)	30 (46.9)	24 (37.5)	1 (1.6)

From here on we used a 10% threshold for positive staining in the clinical analyses. The association between WHO grades and MG1-4 is indicated by Table 4. In MG3 and MG4 more tumors were of WHO grade 2 and 3.

**Table 4:** Distribution of WHO grade^a^ among the four molecular groups, when positive group assignment was set to ≥10%.

Marker group	Grade 1	Grade 2	Grade 3
**MG1**, n (%)	29 (87.9)	4 (12.1)	0 (0)
**MG2**, n (%)	1 (100)	0 (0)	0 (0)
**MG3**, n (%)	44 (75.9)	12 (20.7)	2 (3.4)
**MG4**, n (%)	26 (74.3)	9 (25.7)	0 (0)

a Classified according to WHO classification used at time of surgery (2000, 2007 or 2016).

**Fig. 2:**
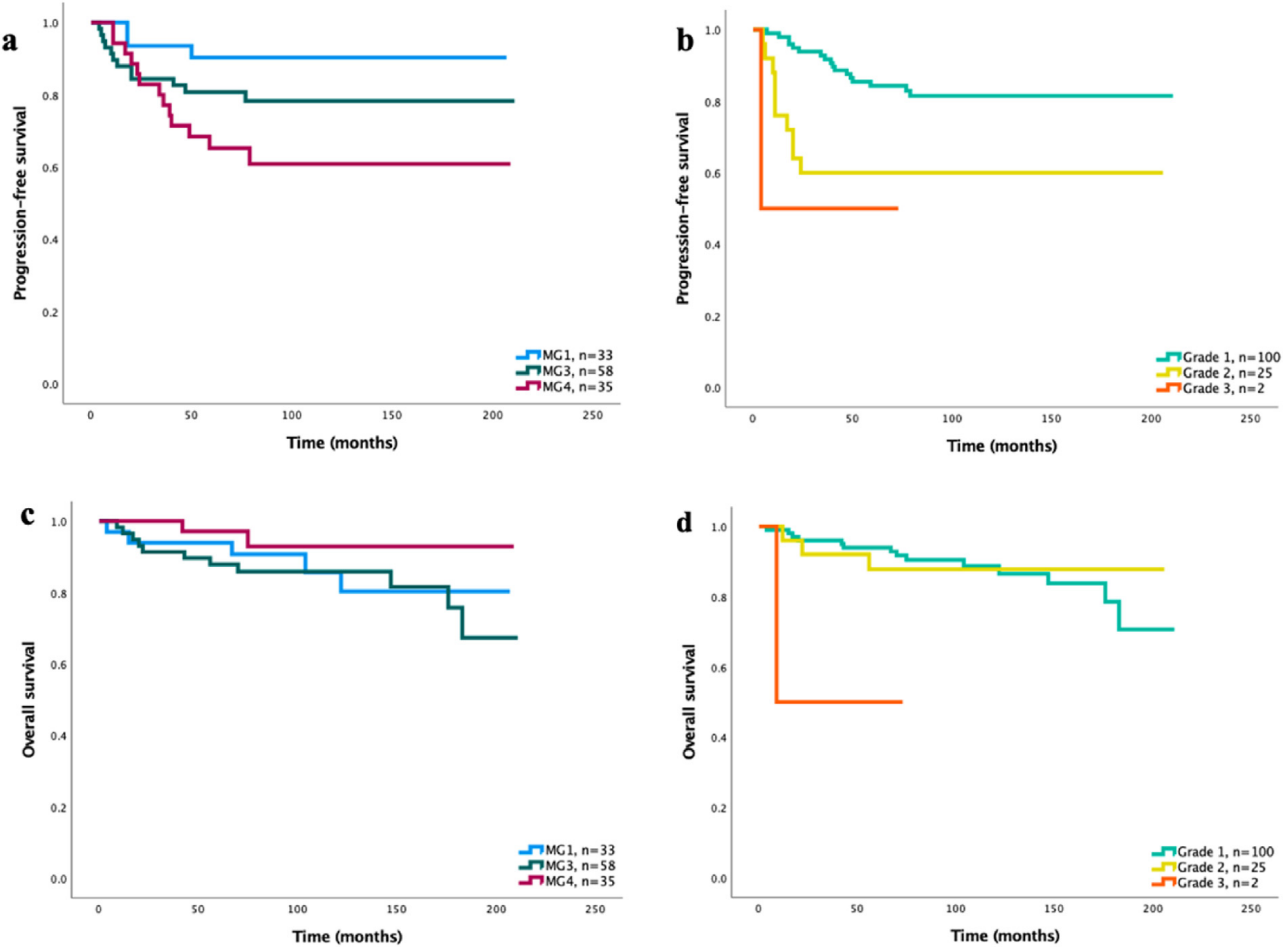
**a)** PFS amongst molecular group 1, 3 and 4∗, n ​= ​126 (p ​= ​0.032). **b)** PFS amongst WHO grade 1–3∗∗, n ​= ​127 (p ​= ​0.005). **c)** OS amongst molecular group 1, 3 and 4∗, n ​= ​126 (p ​= ​0.302). **d)** OS amongst WHO grade 1–3∗∗, n ​= ​127 (p ​= ​0.033). ∗MG2 excluded from analysis due to only one patient assigned to this group. **∗∗** Classified according to WHO classification used at time of surgery (2000, 2007 or 2016).

**Fig. 3:**
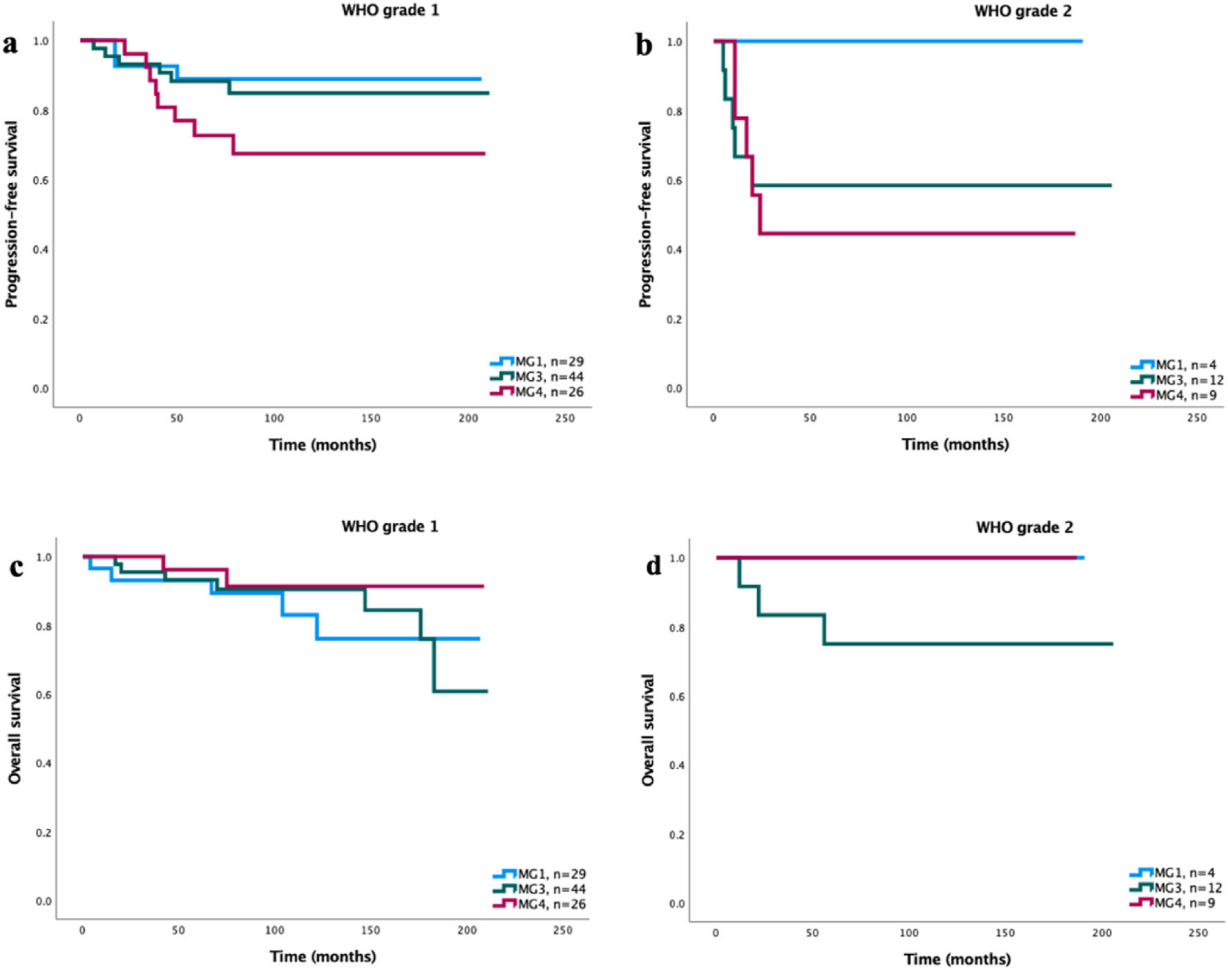
**a)** PFS amongst molecular group 1, 3 and 4∗ when stratified for tumors with WHO grade 1, n ​= ​99 (p ​= ​0.137). **b)** PFS amongst molecular group 1,3 and 4∗ when stratified for tumors with WHO grade 2, n ​= ​25 (p ​= ​0.269). **c)** OS amongst molecular group 1, 3 and 4∗ when stratified for tumors with WHO grade 1, n ​= ​99 (p ​= ​0.587). **d)** OS amongst molecular group 1,3 and 4∗ when stratified for tumors with WHO grade 2, n ​= ​25 (p ​= ​0.177). ∗MG2 excluded from analysis due to only one patient assigned to this group.

**Fig. 4:**
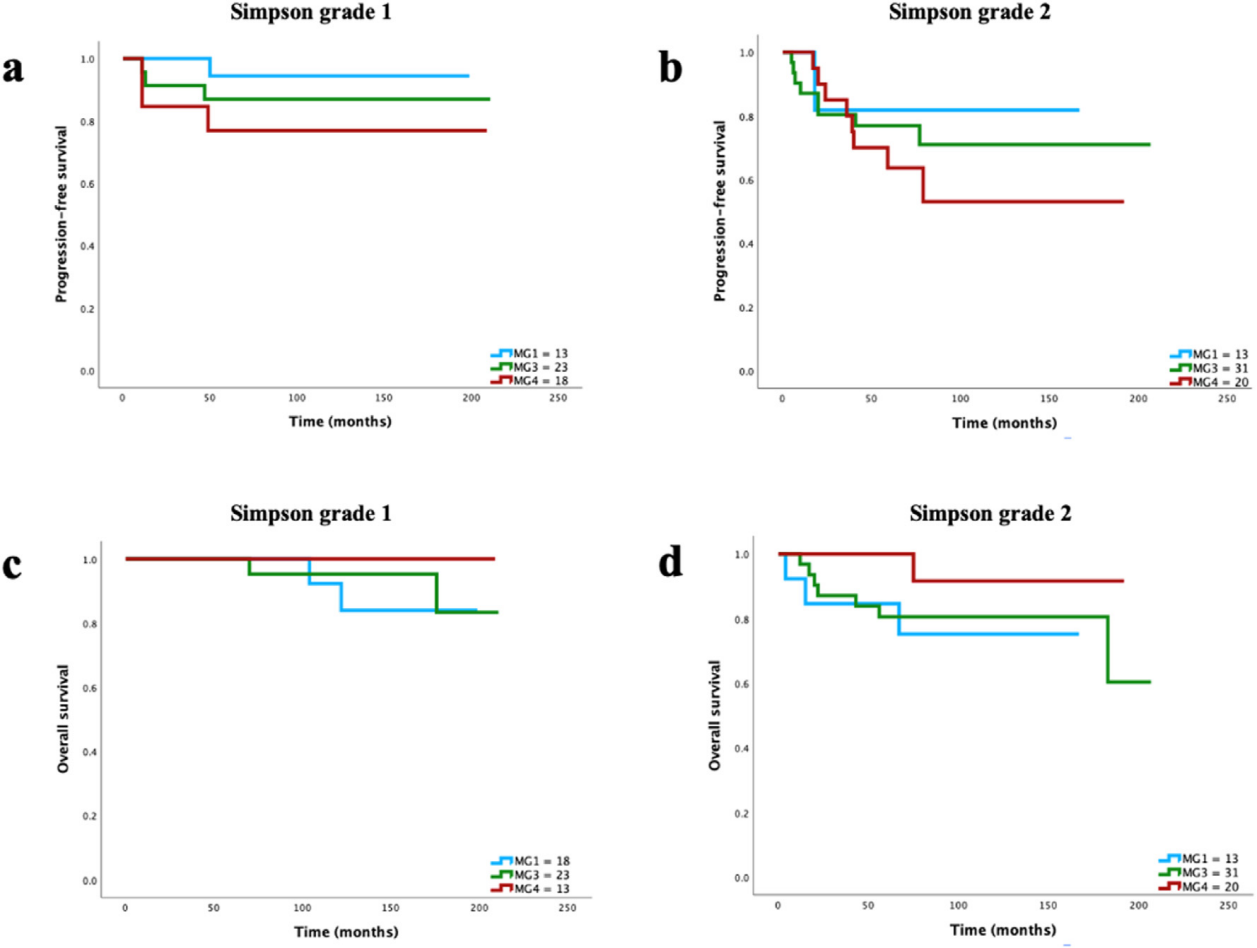
**a)** PFS among molecular group 1, 3 and 4∗ when stratified for tumors with Simpson grade 1 ​at resection (p ​= ​0.34), **b)** PFS among molecular group 1, 3 and 4∗ when stratified for tumors with Simpson grade 2 ​at resection (p ​= ​0.49) **c)** OS among molecular group 1, 3 and 4∗ when stratified for tumors with Simpson grade 1 ​at resection (p ​= ​0.6), **d)** OS among molecular group 1, 3 and 4∗ when stratified for tumors with Simpson grade 2 ​at resection (p ​= ​0.26). ∗MG2 excluded from analysis due to only one patient assigned to this group.

The post hoc analysis of PFS amongst the patients successfully assigned to a molecular group (n ​= ​127) during the second round of analysis is shown in Fig. 2 a-d. In MG2 there was only one patient, hence it was excluded from the survival analysis. Before end of follow up, three patients (9.1%) in MG1, 12 (20.7%) in MG3 and 13 (37.1%) in MG4 had tumor progression, with a significant difference between groups in PFS (Fig. 2a, log-rank p ​= ​0.032). Similarly, the WHO grading had significant impact on PFS (Fig. 2b, log rank p ​= ​0.005). Within the molecular groups, five patients (15.2%) in MG1, 11 patients (19.0%) in MG3 and two patients (5.7%) in MG4 were deceased at end of follow up. OS did not significantly differ between the molecular groups (Fig. 2c, log rank p ​= ​0.302). OS was also studied based on WHO grade as described above, where a significant difference between groups was demonstrated (log rank p ​= ​0.033). When stratifying per WHO grade, neither PFS nor OS differed significantly between the molecular groups (PFS: p ​= ​0.137 vs. p ​= ​0.269, OS: p ​= ​0.587 vs. p ​= ​0.177; see Fig. 3 a-d). Since extent of resection is a crucial modifiable risk-factor we also performed a post-hoc analysis in cases undergoing Simpson grade 1 and 2 resection. Simpson grade 3 and 4 tumors were not analyzed due to too few cases. When stratified for Simpson grade 1 and 2, there was no significant difference in neither PFS nor OS among the different molecular groups (Fig. 4 a-d).

## 4 Discussion

The primary aim of this study was to investigate the replicability and prognostic capability of IHC makers proposed by Nassiri et al. (2021) in a larger cohort of patients with relevant clinical annotation. Studying tissue samples from a clinical database of patients with meningiomas undergoing primary surgery at our hospital, we found that 73.8% of tumor samples could initially not be assigned to either one of the four molecular groups. Using an arbitrary cut-off at <10% (based upon our initial groups), the distribution of patients successfully annotated to a molecular group turned out to be more favorable, but the assigned meningioma group did not clearly outperform or refine WHO grading in terms of prognostication.

One concern when developing new grading systems is the feasibility of implementation in academic and community settings, where accessibility and ease of interpretation are critical. Simple IHC-based diagnostics, similar to the IDH1 R132H antibody stepwise approach for *IDH* mutation, would be desirable - especially for meningioma - due to its speed in diagnostics and usefulness in settings with less resources (Louis et al., 2016, 2020, 2021; Weller et al., 2021). We were therefore encouraged by MG1-MG4 classification, where four new IHC markers were suggested with seemingly clinical useful properties (Nassiri et al., 2021). However, the IHC based classification was not clinically useful in our cohort. This may have been caused by lack of clarity how the results should be interpreted if one sample stained for several markers, and specifically if a low proportion of immunopositive cells should be considered enough for cataloging into the suggested groups. To overcome this problem, we performed two sets of analyses – the first with a simple cut-off with “any positive staining” considered positive. This approach, however, provided annotation to a molecular group in just above 25% of patients. When using a ≥10% inclusion cut-off, successful annotation was just above 50%. Unfortunately, a simple all-or-nothing approach failed, and the markers were not mutually exclusive.

Methodological differences, such as the use of different antibodies for detection of S100b and MCM2, between our study and the study by Nassiri et al. (2021), may explain some of the divergent results. However, it is clear from our study that further work to clarify method and/or set levels for cut-off is needed before these markers can provide clinical value. Another factor important to mention in antibody-based studies is the need for proper antibody validation, to ensure that the staining observed corresponds to the actual protein expression, avoiding cross-reactivity and unspecific antibody binding. In the present investigation, the antibodies were quality-assessed using stringent criteria according to the International Working Group for Antibody Validation (Uhlen et al., 2016; Sivertsson et al., 2020) and the optimal dilution was chosen based on known positive and negative control tissues. While this does not necessarily ensure that deviations in staining patterns would had been observed if other antibodies towards the same proteins were used, it reduces the risk of false interpretations of the protein expression patterns caused by the antibodies.

It has previously been shown that the WHO classification of meningiomas as of now has moderate prognostic capababilities (Patel et al., 2019; Sahm et al., 2017). In recent years, several new methods for classifying meningiomas have been proposed, many of them seemingly superior to WHO grade (Maas et al., 2021; Nassiri et al., 2019; Choudhury et al., 2022). Using the cut-off of 10% positively stained cells, we found that both among the molecular groups and WHO grades there was a significant difference in terms of progression-free survival. When stratifying for WHO grade 1 and 2 tumors, there was no significant difference between either PFS or OS. Therefore, we can conclude that the molecular markers used did not add any apparent value beyond WHO grading in our clinical cohort. The need for new, more reliable methods of predicting tumor behavior and guiding treatment and follow-up regimens remains. Interestingly, the results on PFS and OS show that the patients of group MG1 seem to do better in terms of tumor control, although they die earlier. Paradoxically, the patients of group MG4 show the exact opposite pattern, with poorer tumor control but longer survival. To find out whether age could be a contributing factor we compared age between the groups, but no clear differences in mean age were found (MG1 57.9 years vs MG4 56.0 years, results not shown).

Strengths of this study are long follow-up of patients with complete clinical data, and a well-characterized patient cohort, comparable to the one of Nassiri et al. (2021)*,* that is enriched with higher grade tumors. One major limitation, but also an important finding of the study, was the difficulty in interpretation of the stained tumor samples in the TMAs. Even though it was obvious that some tumors samples had stronger/more widespread staining whilst other had weaker/more scarce staining, there were no clear ways of determining whether a patient could be slotted into either of the groups even if it stained for several of the markers. One might assume that the marker with strongest or most widespread staining should be that of choice when grouping the patients, or a defined cut-off value of amount and intensity of staining required for positivity, but this should be further investigated and is out of the scope of the present paper. The original article by Nassiri et al. (2021) used, besides annotation by blinded neuropathologist, unbiased digital quantification of each protein marker. This method was not used in our replication, but instead three observers aided in the annotation. Each annotation was double checked by two observers as to control for coherence in the assessments, and any differences were solved through discussion with a neuropathologist. Another important limitation to consider is that this paper does not repeat the molecular classification study as performed by Nassiri et al. (2021) in their original paper, but try to clinical establish the usefulness of the proposed IHC-markers to assign meningiomas in the relevant subgroups. However, based upon our results the IHC-markers proposed is not ready for clinical implementation, and future work to externally validate their association with meningioma biology is encouraged.

## 5 Conclusion

We have studied a proposed new method of classifying meningiomas into groups using IHC markers in a well-defined clinical cohort. In our material and in our hands, the IHC based classification in its present form lacks clinical applicability primarily due to lack of exclusivity of markers, with many samples being positive for two or more markers. In a post-hoc analysis we saw significant differences in PFS related to both the IHC assigned meningioma groups, but the IHC assigned group neither significantly outperformed or refined the WHO grading.

## Funding

The study was financed by grants from the Swedish state under the agreement between the Swedish Government and the county councils, the ALF-agreement (A.S.J; ALFGBG-965622, A.S; ALFGBG-965033).

## Authors’ contributions

O.N, A.S.J and A.C planned the study design. O.N and A.L performed the clinical and radiological data collection. A.S.J and A.S provided material support. Tissue microarray construction and staining was performed by C.L, A.D, and T.O.B. The immunohistochemical analysis was performed by O.N, A.L, T.O.B and A.D. O.N, A.S.J and A.C wrote the manuscript and were involved in the submission process. O.N, A.L, A.D, T.O.B, A.S, A.S.J and A.C revised the manuscript. All authors did a final revision of the manuscript and have approved the submitted version.

## Declaration of competing interest

The authors declare that they have no known competing financial interests or personal relationships that could have appeared to influence the work reported in this paper.
