# Supplementary material for: Meningioma classification by immunohistochemistry: A replicability study
Source: Brain Spine. 2022 Dec 27;3:101711. doi: 10.1016/j.bas.2022.101711 (PMC9845417; doi:10.1016/j.bas.2022.101711)
Supplement: Multimedia component 1 [file mmc1.docx]

**Supplementary Table**. Antibodies used for immunohistochemical detection of S100b, SCGN, ACADL and MCM2 in the current study compared to the publication by Nassiri *et al.*

| **Antibodies used** | **Näslund *et al*.** |  | **Nassiri *et al*.** |  |
| --- | --- | --- | --- | --- |
| **Anti-S100b** | HPA015768, Atlas Antibodies 1:5000 | Polyclonal | 701340, ThermoFisher, 1:100 | Monoclonal |
| **Anti-SCGN** | HPA006641, Atlas Antibodies, 1:500 | Polyclonal | HPA006641, Sigma, 1:500 | Polyclonal |
| **Anti-ACADL** | HPA011990, Atlas Antibodies, 1:100 | Polyclonal | HPA01199o, Sigma, 1:200 | Polyclonal |
| **Anti-MCM2** | MCA1859, Bio-Rad Antibodies, 1:200 | Monoclonal | 12079S, Cell signaling, 1:200 | Monoclonal |
